# Prefrontal dysfunction associated with a history of suicide attempts among patients with recent onset schizophrenia

**DOI:** 10.1038/s41537-020-00118-z

**Published:** 2020-10-30

**Authors:** Jun Matsuoka, Shinsuke Koike, Yoshihiro Satomura, Naohiro Okada, Yukika Nishimura, Eisuke Sakakibara, Hanako Sakurada, Mika Yamagishi, Katsuyoshi Takahashi, Yoichiro Takayanagi, Kiyoto Kasai

**Affiliations:** 1 Department of Neuropsychiatry, Graduate School of Medicine, The University of Tokyo, Bunkyo-ku, Tokyo, 113-8655 Japan; 2 UTokyo Institute for Diversity & Adaptation of Human Mind (UTIDAHM), Meguro-ku, Tokyo, 153-8902 Japan; 3 The International Research Center for Neurointelligence (WPI-IRCN) at The University of Tokyo Institutes for Advanced Study (UTIAS), Bunkyo-ku, Tokyo, 113-8655 Japan; 4 Tokyo Metropolitan Matsuzawa Hospital, Setagaya-ku, Tokyo, 156-0057 Japan; 5 Tokyo Metropolitan Ohtsuka Hospital, Toshima-ku, Tokyo, 170-8476 Japan; 6 Department of Neuropsychiatry, University of Toyama Graduate School of Medicine and Pharmaceutical Sciences, Toyama, 930-0194 Japan

**Keywords:** Schizophrenia, Psychosis

## Abstract

Suicide is a major cause of death in patients with schizophrenia, particularly among those with recent disease onset. Although brain imaging studies have identified the neuroanatomical correlates of suicidal behavior, functional brain activity correlates particularly in patients with recent-onset schizophrenia (ROSZ) remain unknown. Using near-infrared spectroscopy (NIRS) recording with a high-density coverage of the prefrontal area, we investigated whether prefrontal activity is altered in patients with ROSZ having a history of suicide attempts. A 52-channel NIRS system was used to examine hemodynamic changes in patients with ROSZ that had a history of suicide attempts (*n* = 24) or that lacked such a history (*n* = 62), and age- and sex-matched healthy controls (*n* = 119), during a block-design letter fluency task (LFT). Patients with a history of suicide attempts exhibited decreased activation in the right dorsolateral prefrontal cortex compared with those without such a history. Our findings indicate that specific regions of the prefrontal cortex may be associated with suicidal attempts, which may have implications for early intervention for psychosis.

## Introduction

Suicide accounts for approximately 5% of deaths among patients with schizophrenia^1^. Schizophrenia is associated with a 10-fold increase in the risk of suicide relative to that observed in the general population^2^, and this risk is especially high in patients with recent disease onset^3,4^. Furthermore, patients with schizophrenia tend to select highly lethal methods of suicide^5,6^, possibly due to positive symptoms or increases in impulsivity^7,8^.

Neuroimaging studies have identified the neural correlates of suicidal behavior in patients with schizophrenia. For instance, a study reported that patients with schizophrenia who have attempted suicide exhibit increases in right amygdala volume relative to that observed in patients without a history of suicide attempts^9^. Another structural magnetic resonance imaging (MRI) study reported that the volumes of the left superior temporal gyrus and orbitofrontal cortex (OFC) are smaller in patients with schizophrenia who have attempted suicide than in those who have not^10^. Patients with schizophrenia with suicidal behavior show cortical thinning in the right dorsolateral prefrontal cortex (DLPFC) and the superior temporal cortex compared to non-suicidal patients^11^. A previous functional MRI (fMRI) study reported that the right DLPFC may play a role in self-harm and suicidal thinking in patients with schizophrenia^12^. Based on these findings, we hypothesized that functional abnormalities in the PFC and temporal cortex, as measured with functional near-infrared spectroscopy (fNIRS), underly suicidal behavior among patients with schizophrenia.

Multi-channel NIRS is a simple, non-invasive neuroimaging modality that can measure changes in oxy- and deoxy-hemoglobin levels in the cortex^13^. NIRS detection of hemoglobin signals has been reported to correlate well with fMRI of blood-oxygenation-level-dependent signals^14^. Recently, NIRS has been used to examine brain changes in patients with psychiatric disorders^15^.

Among patients with major depressive disorder, a history of suicide attempts was associated with decreases in the hemodynamic response in the left dorsolateral prefrontal cortex (DLPFC) during a letter fluency task (LFT)^16^. Moreover, decreased activity in the right DLPFC was associated with aggression and that in the left DLPFC, OFC and temporal cortex with impulsivity^16^. However, it remains unclear whether brain activity as measured using NIRS reflects the predisposition to suicide in patients with schizophrenia. Such investigations should particularly target patients with recent-onset schizophrenia (ROSZ), since the risk of suicide is higher in the earlier stages. Therefore, in the present study, we utilized multi-channel NIRS to investigate whether a history of suicide attempts is associated with functional alterations in the brains of patients with ROSZ.

## Results

### Participant characteristics

Premorbid IQ values were significantly higher in the healthy control group than in the schizophrenia group. PANSS positive and negative symptom scores were significantly lower in the SA+ group than in the SA− group. However, no significant differences in task performance were observed among the groups (Table 1).

**Table 1:** Demographic characteristics.

	SZ (*n* = 86)						Controls (*n* = 119)			
	SA+ (*n* = 24)		SA− (*n* = 62)		SA+ vs SA−				SZ vc Controls	
	Mean	SD	Mean	SD	*P*-value	df	Mean	SD	*P*-value	df
*n* (male/female)	12/12		34/28		0.69^a^	1	68/51		0.60^a^	1
Age (y)	25.4	6.6	25.9	7.3	0.77	84	26.5	5.1	0.40	203
Pre morbid IQ	101.6	13.2	101.7	21.1	0.99	84	109.2	8.3	<0.01	198
LFT score	12	4.6	12.5	5.2	0.69	84	13.3	3.3	0.13	203
PANSS										
Positive	12.7	3.3	15.3	4.5	0.02	69				
Negative	17.5	5.5	21	7.1	0.05	69				
General psychopathology	32.9	8.3	35.1	8.8	0.33	69				
PANSS five-factor model										
Positive symptoms	9.9	3.2	12.0	3.6	0.02	69				
Negative symptoms	19.9	6.0	23.3	8.6	0.09	69				
Disorganization symptoms	9.3	2.3	10.9	3.6	0.06	69				
Excitement	6.0	2.5	6.1	2.1	0.95	69				
Emotional distress	9.7	3.8	9.3	3.6	0.72	69				
GAF	45.4	10.7	42.5	11.2	0.31	73				
Age at onset (y)	24.1	6.6	24.5	7	0.80	84				
DUP (w)	26.1	49.8	32.6	53.5	0.61	82				
DOM (m)	8.7	14.5	10.4	15.2	0.66	82				
CP (mg)	555.7	447.4	558.3	800.6	0.99	83				
Diazepam (mg)	12.9	12.6	8.2	8.8	0.11	80				
Biperiden (mg)	2.4	4	1.7	2.2	0.32	82				

^a^We did chi-squared test, and for the other metrics, we took *t*-test.

*CP* chlorpromazine, *DOM* duration of medication for psychosis, *DUP* duration of untreated psychosis, *GAF* Global Assessment of Functioning, *LFT* Letter Fluency Task, *PANSS* Positive and Negative Syndrome Scale, *SA+* Suicide attempt+, Suicide attempt, *SZ* Schizophrenia.

### Correlation between changes in [Oxy-Hb] and participant characteristics

We observed trend-level positive correlations between changes in [Oxy-Hb] and premorbid IQ score for the following channels: ch4, ch14, ch18, ch26, ch36, ch46, ch47 (*r* = 0.214–0.294, *p* = 0.0080–0.0046, FDR-uncorrected). In addition, we observed trend-level negative correlations between changes in [Oxy-Hb] and LFT scores for the following channels: ch8, ch26–28 (*r* = −0.276–−0.216, *p* = 0.011–0.048, FDR-uncorrected). We also observed a trend-level positive correlation between changes in [Oxy-Hb] and PANSS positive symptom scores for ch15 (*r* = 0.266, *p* = 0.027, FDR-uncorrected).

### Mean changes in [Oxy-Hb] during the task period

**Fig. 1:**
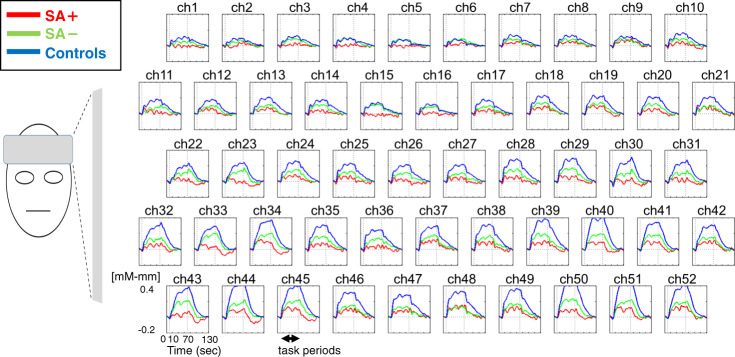
Grand-averaged waveforms during the letter fluency task in the three groups. The 52 measurement areas are labeled ch1–52 from the right-superior to the left-inferior. The task period is shown between the vertical dash lines and also indicated by a double arrow.

Changes in [Oxy-Hb] for the three groups are presented in Fig. 1. We identified significant main effects at 34 channels (ch11, ch13–15, ch17, ch21, ch23, ch24–29, ch31–36, ch38–52; *F* = 3.20 to 17.59, *p* < 0.0024, FDR-corrected) distributed over the front-temporal cortical regions.

**Fig. 2:**
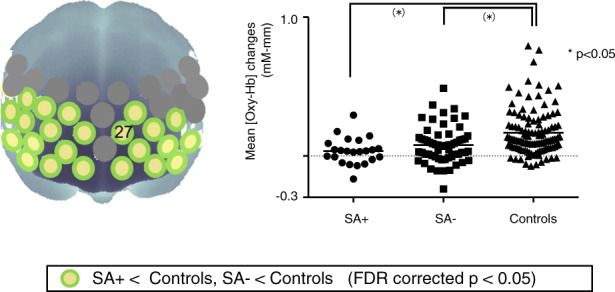
Three-dimensional cerebral maps of different hemodynamic response patterns during the task periods. Left: Channels exhibiting significant changes in mean [Oxy-Hb] during the task period, as determined using post hoc Tukey’s honest significant difference (HSD) tests. Right: Dot plots of mean changes in [Oxy-Hb] at a representative channel (ch27).

Post hoc Tukey HSD tests revealed that activation in these 34 channels was significantly lower in the schizophrenia group (SA+ and/or SA−) than in the control group (Fig. 2). The absolute value of Cohen’s d ranged from 0.03 to 0.77 between the schizophrenia groups.

### Relationship between history of suicide attempts and changes in [Oxy-Hb]

**Fig. 3:**
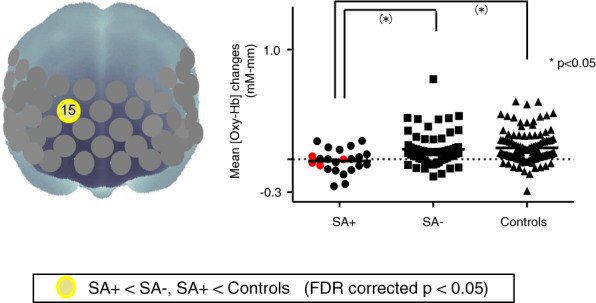
Three-dimensional topographic maps during the task periods showing a significant difference between the SA+ and SA− groups. Left: Ch15 corresponds to the right DLPFC. Right: Dot plots of mean changes in [Oxy-Hb] at ch15. The red plots represent the SA+ after NIRS. SA+: suicide attempt; SA−: non-suicide attempt.

**Fig. 4:**
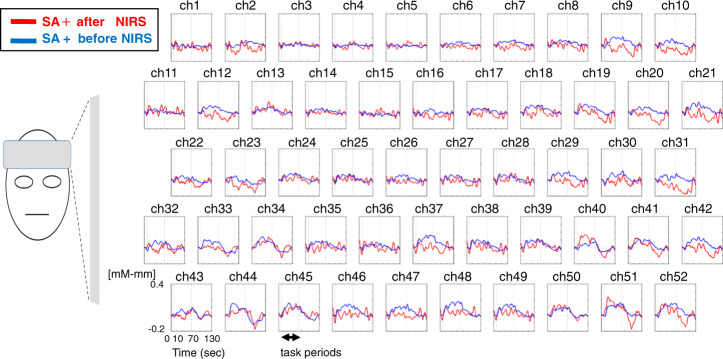
Grand-averaged waveforms during the letter fluency task in the SA+ after NIRS group and SA+ before NIRS group. SA+: suicide attempt; SA−: non-suicide attempt.

Significant differences in brain activity were observed between the SA+ and SA− groups at one channel in the right DLPFC (ch15, *F* = 6.918, *p* = 0.001, FDR-corrected; Fig. 3). We observed trend-level positive correlations between changes in [Oxy-Hb] and the positive symptom factor score for ch15 (*r* = 0.298, *p* = 0.013, FDR-uncorrected). The association between changes in [Oxy-Hb] and history of suicide attempts remained significant after controlling for the positive symptom factor score in a multiple regression analysis (*R*^2^ = 0.253, adjusted *R*^2^ = 0.181, *β* = 0.293, *t* = 2.547, *p* = 0.013) significant differences in brain activity were observed between the SA+ after NIRS group and SA+ before NIRS group (Fig. 4).

## Discussion

The present study utilized NIRS to investigate the association between hemodynamic dysfunction in the prefrontal cortex and suicide attempts in patients with ROSZ. Our results indicated that patients with a history of suicide attempts exhibited lower activation in the right DLPFC than did those without a history of suicide attempts, which was not solely attributed to differences in symptom severity.

In the present study, brain activity in the right DLPFC during the LFT was significantly lower in the SA+ group than in the SA- group. Previous researches have revealed that among patients with schizophrenia those with a history of suicide attempts are more impulsive than those without it^9,17^. The right DLPFC is among the brain regions responsible for impulse control, along with the OFC and VLPFC^18,19^. Decreased volumes in the fronto-polar cortex, upper temporal cortex, anterior cingulate cortex^20^, and OFC^21^ have been associated with impulsivity among patients with schizophrenia. Previous functional imaging studies have also reported dysfunction in the right DLPFC during an AX-continuous performance task^22^ and in the right DLPFC and left VLPFC during the Stroop task^23^. Our previous NIRS study indicated that changes in [Oxy-Hb] in the right DLPFC during a go/no-go task were positively correlated with PANSS excitement scores^24^. These findings suggest an association between prefrontal dysfunction and impairments in impulse control in patients with schizophrenia. Taken together, the lower activity in the right DLPFC may underlie the basis for impulsivity in schizophrenia, leading to a higher risk for suicidal attempts. The excitement component of the PANSS five-factor model was measured as a surrogate for impulsivity, although specific neuropsychological tests for impulsivity would be helpful to further explain the association between the deficiency in NIRS activation and inhibition control.

The mean interval between NIRS measurement and suicidal behavior was 46.2 ± 68.5 weeks. The interval was not associated with a hemodynamic response at ch15 (*p* = 0.098) or clinical symptoms in the PANSS five-factor model (*p* = 0.399–0.838). A possible explanation for these results is that the brain function abnormality found in this study reflects some trait component of the risk factors for suicidality.

We used LFT as a cognitive task in accordance with the multicenter study we took part in^25^. A previous meta-analysis suggested that the category fluency test (CFT) is a cognitive task that is preferable for elucidating brain dysfunction associated with a history of suicide attempt in patients with mood disorders^26^. A neuroimaging study using single photon emission computed tomography for patients with major depressive disorder found that those with suicidal behaviors showed significantly reduced perfusions in the left inferior frontal gyrus, right inferior parietal lobule, and bilateral anterior cingulate cortex during CFT^27^. This finding contrasts that in our study. This inconsistency could be due to the heterogeneity in the patient groups (mood disorders vs. schizophrenia) or due to the difference in the cognitive task used (CFT vs. LFT). While neither the LFT nor the CFT are cognitive tasks specifically relevant to inhibition control, the LFT might be more suitable for illuminating functional abnormalities in patients with schizophrenia because previous studies reported that [Oxy-Hb] changes in patients with schizophrenia during LFT were evident across broader prefrontal areas than during the CFT^28,29^.

The present study possesses some limitations of note. First, many patients were taking medication at the time of NIRS measurement, which may have affected brain function and suicidal ideation. However, previous studies have repeatedly reported that there is no association between medication doses and NIRS activity in patients with schizophrenia^13^. Furthermore, medication doses did not significantly differ between the SA+ and SA− groups in our study. Second, although NIRS activity is not significantly different between four SA+ participants who committed suicide after NIRS measurement and 20 SA+ participants who committed suicide before NIRS measurement, further prospective studies are required to determine whether the brain activity in the DLPFC has a predictive value for subsequent suicidal behavior. Third, since we did not use a structured method to obtain information regarding suicide attempts, patient histories may have been somewhat inaccurate. Fourth, our sample size was relatively small. Among the 34 channels in which activation in the SA+ and/or SA− groups was significantly smaller than in the control group, the Cohen’s d for the [Oxy-Hb] difference between the SA+ and SA− groups was greater than 0.5 for 8 channels (ch14, 15, 28, 29, 35, 39, 43, 50). Increasing the sample size might identify further brain regions associated with suicidal behavior. Fifth, we did not obtain data related to trauma history in our participants. Since trauma is associated with both subsequent suicidal behavior^30^ and prefrontal dysfunction^31^, this factor may mediate the associations found in our study.

The present multi-channel NIRS study revealed an association between LFT-induced hemodynamic impairments in the right DLPFC and a history of suicide attempts among patients with ROSZ. These findings may have implications for biological evidence-based intervention for early stages of psychosis. A further study is needed to investigate the long-term outcomes of patients with ROSZ.

## Methods

### Participants

The medical charts of the patients with schizophrenia who have NIRS measurement data were reviewed retrospectively at the University of Tokyo Hospital and the Tokyo Metropolitan Matsuzawa Hospital. Eighty-six patients with ROSZ and 119 age-, sex-, and task performance-matched healthy controls participated in the current study. All patients were recruited from outpatient and inpatient units at the University of Tokyo Hospital and the Tokyo Metropolitan Matsuzawa Hospital from April 1, 2004, to February 28, 2015. This study was approved by The Research Ethics Committee of the Faculty of Medicine of The University of Tokyo (approval No. 630, 2226, 3202), and by The Ethical Committee of The Tokyo Metropolitan Matsuzawa Hospital (approval No. 20). An opt-out policy was applied for collecting clinical data (No. 3349). All participants gave written informed consent in accordance with the Declaration of Helsinki before their participation in the study.

Similar to our previous study^32^, ROSZ was defined as follows: age ranging from 15–40 years and continuous psychotic symptoms beginning within the past 60 months. Diagnoses of schizophrenia were confirmed by well-trained psychiatrists based on detailed clinical interviews involving participants and their family members, in accordance the criteria outlined in the Diagnostic and Statistical Manual of Mental Disorders, Fourth Edition^33^.

Levels of functioning and symptoms in patients with schizophrenia were evaluated using the modified Global Assessment of Functioning (GAF)^34,35^ and the Positive and Negative Syndrome Scale (PANSS)^36^. Chlorpromazine-, diazepam-, and biperiden-equivalent doses were calculated in patients taking antipsychotics, benzodiazepines, and/or antiparkinsonian drugs, respectively^37^. Premorbid intelligence quotients (IQs) for patients with schizophrenia and IQs for the control group were estimated using the 25-item Japanese version of the National Adult Reading Test^38,39^.

In both groups, exclusion criteria were as follows: neurological diseases, a history of loss of consciousness for more than 5 min due to traumatic head injury, a history of electroconvulsive therapy, low premorbid IQ (below 70), and a previous history of alcohol abuse or addiction. In the control group, the Mini-International Neuropsychiatric Interview (MINI)^40^ was used to rule out psychiatric disorders and exclude participants whose first-degree relatives had been diagnosed with psychotic disorders.

Information regarding previous suicide attempts was obtained via interviews with all 86 patients and their family members at the time of NIRS assessment. In total, 20 patients had a history of suicide attempts prior to NIRS assessments. In addition, suicide attempts after the NIRS measurement were examined in 62 participants whose medical records were available. Among them, four patients attempted suicide after NIRS measurement during the mean ± SD follow-up period of 35.0 ± 28.6 months. Thus, 24 patients were classified into the suicide attempt (SA+) group, while the remaining 62 patients were classified into the non-suicide attempt (SA−) group. The temporal distance between suicide attempts and NIRS measurements is shown in Table 2.

**Table 2:** Details of suicide attempts.

Case	Suicide methods	Interval (week)
1	Hanging	23
2	Hanging	276
3	Cutting with nerve injury	85
4	Overdose	4
5	Overdose	136
6	Hanging	8
7	Cutting	45
8	Hanging	16
9	Hanging	10
10	Overdose	8
11	Overdose	17
12	Hanging, Overdose	5
13	Overdose	N/A
14	Overdose	40
15	Cutting, Overdose	5
16	Hanging, Overdose	6
17	Drowning	15
18	N/A	N/A
19	Jumping	N/A
20	Cutting	N/A
21	Cutting	N/A
22	Overdose	125
23	Stabbing	7
24	Overdose	46

Cases 1, 4, 8, and 10 attempted suicide after NIRS measurement date. The interval was calculated from the NIRS measurement date and the suicide attempt date.

Abbreviation: N/A, not available.

### NIRS apparatus

**Fig. 5:**
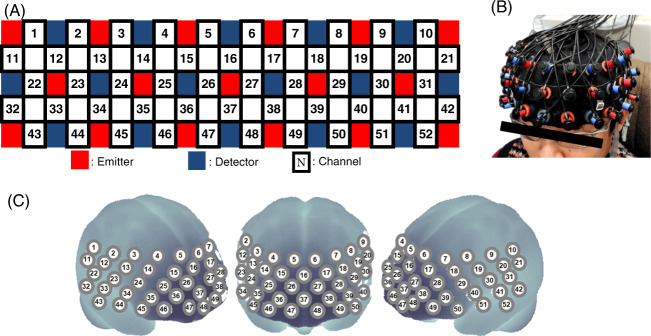
Probe settings and cortical regions assessed using a 52-channel near-infrared spectroscopy (NIRS) system. **a** Arrangement of emitter and detector probes and definitions for each channel. **b** Probe settings over the frontal regions. The image of person is one of the authors. The consent for use of this image was obtained. **c** Anatomical regions corresponding to each channel.

A 52-channel NIRS instrument (ETG-4000; Hitachi Medical Co. LTD., Tokyo, Japan) was used to measure relative changes in oxygenated and deoxygenated hemoglobin using 695 nm and 830 nm wavelengths of near-infrared light. The sampling rate of the NIRS signal was set at 0.1 s. The same device was used in our previous studies^41^, in which the 16 emitter probes and 15 detector probes were fixed alternately with 3 × 11 thermoplastic shells set with 52 fixed channels (Fig. 5). The distance between pairs of emitter–detector probes was 3.0 cm, and each measurement area between pairs of emitter–detector probes was defined as one channel.

Brain activity was assessed based on oxygenated hemoglobin ([Oxy-Hb]) levels because increased oxygenated hemoglobin is considered indicative of increased cognitive activation. This indication is considered more direct than a decrease in deoxygenated hemoglobin. Importantly, increased oxygenated hemoglobin levels are more strongly correlated with blood-oxygenation-level-dependent signals measured via fMRI^42^.

The lowest 11 probes were located along the Fp1–Fp2 line, in accordance with the international 10–20 system for electroencephalography. This probe arrangement allows for the measurement of hemoglobin changes in regions of the bilateral cortical surface such as the DLPFC, ventrolateral prefrontal cortex (VLPFC), fronto-polar area (FP), and superior temporal cortical surface regions (Fig. 5). A virtual registration method was used to estimate the cortical localization of each channel^14,43^.

After participants were seated comfortably in a chair, the NIRS cap was placed and the probes were attached. The participants were instructed to keep their eyes open and remain relaxed. To minimize motion artifacts during measurement the participants were instructed to avoid physical movements such as head motions and strong chewing.

### Signal preprocessing

[Oxy-Hb] signals during the task period were averaged using a linear fitting method between those during the last 10 s of the pre-task period and the last 5 s of the post-task period. The moving average with a 5 s window was applied to remove motion artifacts. An artifact rejection method for each channel was adopted using the algorithm published by Koike et al. in 2011^26^ and, if significant artifacts were detected, we excluded the channels from further analyses. Throughout the signal preprocessing, we used mean [Oxy-Hb] during the task period as the brain activity in this study.

### Letter fluency task

Cognitive activation was assessed using an NIRS-adapted 160-s block-design LFT^25,41,44^. Briefly, during the 60-s task period, participants were instructed to state as many Japanese words as possible beginning with the phonological syllable designated by the computer. The task period was divided into three sub-periods, with each consisting of three syllabic stimuli (first: /to/, /a/, or /na/; second: /i/, /ki/, or /se/; third: /ta/, /o/, or /ha/), which were presented in a pseudo-random order and changed every 20 s to avoid silence from the participant. In the 30-s pre-task and 70-s post-task periods, the participant was instructed to simply repeat Japanese vowels (/a/, /i/, /u/, /e/, and /o/) out loud to remove the influence of brain activity associated with speech during the presentation of computer commands. The total number of correct words generated during the task period was considered to reflect task performance.

### Statistical analysis

Pearson’s correlation coefficient was used to investigate relationships between changes in [Oxy-Hb] in each channel and clinical variables including age at measurement, premorbid IQ, LFT scores, GAF scores, and PANSS scores among patients with schizophrenia. A false discovery rate (FDR) method was adopted to correct for multiple comparisons (no more than 5% false positives on average)^45^.

Due to NIRS activity not being significantly different between the four SA+ participants who committed suicide after NIRS measurement and 20 SA+ participants who committed suicide before NIRS measurement (Mann–Whitney, *p* = 0.141–0.938), these participants were treated as one SA+ group. Analyses of variance (ANOVA) were used to compare NIRS activity during the task period among three groups (SA+, SA−, and control groups). The significance of the correlations was examined using the aforementioned FDR method. Post hoc Tukey’s honest significant difference (HSD) tests were used to further evaluate channels exhibiting significant differences in these analyses. To investigate differences between the SA+ group and the SA− group, we also calculated the effect size (Cohen’s d) for channels demonstrating a significant main effect of patient group. For channels exhibiting significant differences in the post hoc Tukey’s HSD test, Pearson’s correlation coefficient was used to assess the relationships between changes in [Oxy-Hb] and a five-factor model of PANSS (the positive symptoms, negative symptoms, disorganization, excitement, and emotional distress) scores^46^. Then, multiple regression analyses were performed using changes in [Oxy-Hb] as the dependent variable and history of suicide attempts (SA+ = 1, SA− = 2), age, sex (male = 1, female = 2), premorbid IQ, LFT score, and a five-factor model of PANSS subscores that were significantly different between the SA+ and SA− groups as independent variables. The differences in NIRS activity due to the violence of the suicide methods used were also examined (Supplementary Table 1). A *P*-*value* (p) < 0.05 was considered significant. All analyses were conducted using SPSS 22.0 (SPSS Inc., Chicago, IL, USA).

### Reporting summary

Further information on research design is available in the Nature Research Reporting Summary linked to this article.

## Supplementary information

Supplementary Table 1

Reporting Summary

## Data Availability

The datasets generated during and/or analyzed during the current study are not publicly available due to ethical codes for this study but are available from the corresponding author on reasonable request with the approval of The Research Ethics Committee of the Faculty of Medicine of The University of Tokyo and The Ethical Committee of The Tokyo Metropolitan Matsuzawa Hospital.
