# Supplementary material for: Prefrontal dysfunction associated with a history of suicide attempts among patients with recent onset schizophrenia
Source: NPJ Schizophr. 2020 Oct 30;6:29. doi: 10.1038/s41537-020-00118-z (PMC7599216; doi:10.1038/s41537-020-00118-z)
Supplement: Supplementary file 1 — Supplementary Table 1 [file 41537_2020_118_MOESM1_ESM.pdf]

Supplementary Table 1. Differences of mean [Oxy-Hb] changes during LFT between violent SA group and non-violent SA group

	violent SA (n = 10)		non-violent SA (n = 13)			
	mean	SD	mean	SD	P value	df
ch01	0.002519	0.070187	-0.00997	0.077151	0.70*	20
ch02	0.015881	0.046937	-0.0264	0.111503	0.28	19
ch03	0.027196	0.067978	-0.0063	0.07714	0.30	20
ch04	0.008101	0.082848	-0.00093	0.075326	0.79	21
ch05	-0.00464	0.090667	-0.0031	0.10642	0.97	21
ch06	0.022363	0.089926	0.010005	0.056859	0.70	20
ch07	0.004323	0.089396	0.064531	0.117997	0.21	19
ch08	0.000551	0.080348	0.078112	0.128818	0.11	20
ch09	0.034389	0.096055	0.05984	0.140763	0.64	19
ch10	0.030133	0.061389	0.020799	0.108643	0.81	20
ch11	0.034074	0.072852	0.019983	0.095574	0.70	21
ch12	0.043147	0.050902	0.036842	0.124444	0.88	21
ch13	0.053919	0.07547	0.035723	0.079569	0.58	21
ch14	0.055391	0.132839	0.006252	0.131701	0.39	21
ch15	-0.01148	0.110904	-0.01447	0.112286	0.95	20
ch16	0.010193	0.095201	0.004152	0.052751	0.86	19
ch17	0.044842	0.103958	0.030694	0.07996	0.72	21
ch18	0.050647	0.112786	0.077789	0.135941	0.62	20
ch19	0.051256	0.117231	0.10232	0.158182	0.40	21
ch20	0.030309	0.139036	0.032832	0.106482	0.96	20
ch21	0.054469	0.059277	0.068648	0.15474	0.79	21
ch22	0.018644	0.190954	0.015327	0.128367	0.96	21
ch23	0.042381	0.145544	-0.03056	0.168188	0.29	21
ch24	0.072667	0.107305	0.042788	0.090103	0.48	21
ch25	0.044016	0.085021	0.050092	0.104601	0.88	21
ch26	0.009818	0.166034	0.027519	0.111402	0.77	20
ch27	0.062724	0.108351	0.014565	0.088963	0.27	20
ch28	0.048358	0.130459	0.04742	0.126655	0.99	21
ch29	0.028473	0.191148	0.070345	0.112532	0.52	21
ch30	-0.01925	0.284543	0.0136	0.146676	0.72	21
ch31	0.029412	0.226174	-0.00079	0.140927	0.70	21
ch32	0.044326	0.207761	0.033044	0.125205	0.87	21

ch33	0.018891	0.243302	0.027301	0.172037	0.92	21
ch34	0.116772	0.151287	0.063481	0.164792	0.43	21
ch35	0.049	0.12574	0.057604	0.112031	0.86	21
ch36	-0.00152	0.218092	0.049951	0.09652	0.45	21
ch37	0.066633	0.11375	0.097489	0.166818	0.64	19
ch38	0.072816	0.218236	0.071912	0.150284	0.99	21
ch39	0.080069	0.116569	0.072031	0.131254	0.88	21
ch40	0.047252	0.237075	0.081578	0.102537	0.64	21
ch41	0.079158	0.257623	0.055077	0.093359	0.77	20
ch42	0.096108	0.146109	0.04367	0.138746	0.39	21
ch43	0.006715	0.309455	0.038289	0.164855	0.76	21
ch44	0.048545	0.314474	0.085633	0.209966	0.74	20
ch45	0.081883	0.192482	0.111988	0.202956	0.73	20
ch46	0.070707	0.207009	0.072228	0.136286	0.98	21
ch47	0.093577	0.130484	0.053156	0.156199	0.53	20
ch48	0.132915	0.154141	0.098906	0.176073	0.64	20
ch49	0.097084	0.221117	0.0892	0.144145	0.92	21
ch50	0.048711	0.228931	0.085632	0.139357	0.64	21
ch51	0.071177	0.244389	0.103783	0.138119	0.69	21
ch52	0.102399	0.230318	0.089326	0.113567	0.86	20

We divided the SA+ group into a violent SA group (hanging, jumping, drowning, and cutting with nerve injury) and non-violent SA group (other methods).
* We took t-test.
Abbreviation: SA, Suicide attempt
