# Supplementary material for: Prefrontal dysfunction associated with a history of suicide attempts among patients with recent onset schizophrenia
Source: NPJ Schizophr. 2020 Oct 30;6:29. doi: 10.1038/s41537-020-00118-z (PMC7599216; doi:10.1038/s41537-020-00118-z)
Supplement: Supplementary file 2 — Reporting Summary [file 41537_2020_118_MOESM2_ESM.pdf]

nature research

Corresponding author(s): Kiyoto Kasai

Last updated by author(s): Sep 3, 2020

Reporting Summary

Nature Research wishes to improve the reproducibility of the work that we publish. This form provides structure for consistency and transparency in reporting. For further information on Nature Research policies, see our Editorial Policies and the Editorial Policy Checklist.

Statistics

For all statistical analyses, confirm that the following items are present in the figure legend, table legend, main text, or Methods section.

n/a	Confirmed	
☐	☒	The exact sample size (n) for each experimental group/condition, given as a discrete number and unit of measurement
☐	☒	A statement on whether measurements were taken from distinct samples or whether the same sample was measured repeatedly
☐	☒	The statistical test(s) used AND whether they are one- or two-sided<br>*Only common tests should be described solely by name; describe more complex techniques in the Methods section.*
☐	☒	A description of all covariates tested
☐	☒	A description of any assumptions or corrections, such as tests of normality and adjustment for multiple comparisons
☐	☒	A full description of the statistical parameters including central tendency (e.g. means) or other basic estimates (e.g. regression coefficient) AND variation (e.g. standard deviation) or associated estimates of uncertainty (e.g. confidence intervals)
☐	☒	For null hypothesis testing, the test statistic (e.g. F, t, r) with confidence intervals, effect sizes, degrees of freedom and P value noted<br>*Give P values as exact values whenever suitable.*
☒	☐	For Bayesian analysis, information on the choice of priors and Markov chain Monte Carlo settings
☒	☐	For hierarchical and complex designs, identification of the appropriate level for tests and full reporting of outcomes
☐	☒	Estimates of effect sizes (e.g. Cohen's d, Pearson's r), indicating how they were calculated

Our web collection on statistics for biologists contains articles on many of the points above.

Software and code

Policy information about availability of computer code

Data collection	No software was used.
Data analysis	SPSS 22.0 (SPSS Inc., Chicago, IL, USA) was used.

For manuscripts utilizing custom algorithms or software that are central to the research but not yet described in published literature, software must be made available to editors and reviewers. We strongly encourage code deposition in a community repository (e.g. GitHub). See the Nature Research guidelines for submitting code & software for further information.

Data

Policy information about availability of data

All manuscripts must include a data availability statement. This statement should provide the following information, where applicable:

- Accession codes, unique identifiers, or web links for publicly available datasets
- A list of figures that have associated raw data
- A description of any restrictions on data availability

The datasets generated during and/or analyzed during the current study are not publicly available due to ethical codes for this study but are available from the corresponding author on reasonable request with the approval of The Research Ethics Committee of the Faculty of Medicine of The University of Tokyo and The Ethical Committee of The Tokyo Metropolitan Matsuzawa Hospital.

Field-specific reporting

Please select the one below that is the best fit for your research. If you are not sure, read the appropriate sections before making your selection.

[x] Life sciences [ ] Behavioural & social sciences [ ] Ecological, evolutionary & environmental sciences

For a reference copy of the document with all sections, see nature.com/documents/nr-reporting-summary-flat.pdf

Life sciences study design

All studies must disclose on these points even when the disclosure is negative.

Sample size	No prior sample size calculations were performed. Because this is an exploratory study, we used the largest sample available in the setting in which we conducted our study.
Data exclusions	Exclusion criteria were as follows: neurological diseases, a history of loss of consciousness for more than 5 min due to traumatic head injury, a history of electroconvulsive therapy, low premorbid IQ (below 70), and a previous history of alcohol abuse or addiction. The exclusion criteria were defined according to our previous studies before recruiting participants for this study.
Replication	No verifying the reproducibility was performed. This is because we did not have a sufficient number of samples to verify the reproducibility.
Randomization	NA, observational study.
Blinding	We did not perform blinding as we would in RCT as this study was an exploratory observational study, although the measurements with near-infrared spectroscopy were conducted by researchers who did not know the clinical information of the study participants.

Reporting for specific materials, systems and methods

We require information from authors about some types of materials, experimental systems and methods used in many studies. Here, indicate whether each material, system or method listed is relevant to your study. If you are not sure if a list item applies to your research, read the appropriate section before selecting a response.

Materials & experimental systems

n/a	Involved in the study	
[x]	[ ]	Antibodies
[x]	[ ]	Eukaryotic cell lines
[x]	[ ]	Palaeontology and archaeology
[x]	[ ]	Animals and other organisms
[ ]	[x]	Human research participants
[x]	[ ]	Clinical data
[x]	[ ]	Dual use research of concern

Methods

n/a	Involved in the study	
[x]	[ ]	ChIP-seq
[x]	[ ]	Flow cytometry
[x]	[ ]	MRI-based neuroimaging

Human research participants

Policy information about studies involving human research participants

Population characteristics	Eighty-six patients with recent-onset schizophrenia and 119 age-, sex-, and task performance-matched healthy controls.
Recruitment	All participants were recruited from outpatient and inpatient units at the University of Tokyo Hospital and the Tokyo Metropolitan Matsuzawa Hospital from April 1, 2004, to February 28, 2015.
Ethics oversight	The Research Ethics Committee of the Faculty of Medicine of The University of Tokyo (approval No. 630, 2226, 3202), and The Ethical Committee of The Tokyo Metropolitan Matsuzawa Hospital (approval No. 20)

Note that full information on the approval of the study protocol must also be provided in the manuscript.
